# Single and Especially Multisport Participation May Increase Physical Fitness in Younger Americans

**DOI:** 10.3390/sports10120202

**Published:** 2022-12-09

**Authors:** Jenny Linker, Bradford Strand, Joe Deutsch, Seungmin Kang, Kelsey Slater, Alaina Driscoll, Ryan McGrath

**Affiliations:** Department of Health, Nutrition, and Exercise Sciences, North Dakota State University, Fargo, ND 58108, USA

**Keywords:** fitness levels, physical education, public health, sport participation

## Abstract

Background: We sought to examine the associations between sports participation and physical fitness in younger Americans. Methods: The analytic sample included 1108 Americans aged 6–15 years. Muscular fitness assessments included handgrip strength, leg extension strength, and plank position. Cardiorespiratory fitness was examined with graded treadmill tests. Results: Youth participating in any sports had increased physical fitness performance for each marker: 1.63 kg (CI: 0.91–2.35) for handgrip strength, 6.23 kg (CI: 3.15–9.32) for leg extension strength, 9.41 lbs. (CI: 4.51–14.31) for plank position held, and 3.02 mL/kg/min (CI: 1.23–4.80) for estimated maximal oxygen uptake. Participants engaging in a single sport had increased performance for most fitness markers: 1.52 kg (CI: 0.67–2.38) for handgrip strength, 5.16 kg (CI: 1.50–8.82) for leg extension strength, 5.98 s (CI: 0.17–11.78) for plank position held, and 2.67 mL/kg/min (CI: 0.58–4.77) for estimated maximal oxygen uptake. Youth engaging in multiple sports exhibited even steeper trends: 1.77 kg (CI: 0.83–2.70) for handgrip strength, 7.63 kg (CI: 3.62–11.65) for leg extension strength, 13.89 s (CI: 7.52–20.27) for plank position held, and 3.50 mL/kg/min (CI: 1.15–5.85) for estimated maximal oxygen uptake. Conclusions: Any sports participation, including single and multiple sports, is linked to increased physical fitness in American youth.

## 1. Introduction

Physical activity participation is well known for promoting physical development and fitness in youth [1]. However, many youth in the United States are not meeting physical activity guidelines, such that less than a quarter of youth aged 6–17 years participate in at least 60 min of moderate-to-vigorous physical activity daily [2]. Similarly, over half of Americans aged 5–11 years engage in long durations of seated television watching or computer usage outside of school, and Americans aged 12–19 years spend over 8 h/day seated [3]. Accordingly, several federal youth initiatives have been launched to stimulate physical activity in the United States such as comprehensive physical education and activity opportunities before, during, and after school [4]. Although the United States Report Card on Physical Activity for Youth graded overall physical activity participation as nearly failing, sport participation has consistently remained a higher scoring indicator for increasing physical activity [5].

Youth sports engagement is encouraged as a mode for increasing lifespan activity adherence and physical health [6], which, in turn, explains why youth sports participation is linked to muscular and cardiovascular fitness [7,8]. While sports participation seems like a logical activity for elevating physical activity participation, there is currently inadequate evidence to conclude if the association between physical activity and health differs by physical activity domain in children and adolescents [9]. Therefore, research is warranted to examine how domain-specific physical activities, such as sports, may help to improve health [9].

There is also a need to further examine the determinates associated with both single and multisport participation. Skillset, injury, and psychosocial factors have been compared and contrasted [10,11,12,13,14,15], but differences in physical attributes (e.g., physical fitness) may also exist. For example, youth multisport participation may facilitate improved coordination [16]. Moreover, multisport participants interestingly performed better in shuttle run, push-up, and running tests compared to single sport participants in persons aged approximately 11 years [16]. Given that coordination and human performance may differ between single and multisport youth participants, characteristics related to fitness and health may likewise be dissimilar. Understanding how single and multisport participation may influence physical fitness may help physical educators, coaches, parents/guardians, and youth optimize the health benefits derived from sport participation and inform choices to specialize in one sport or engage in multiple sports. This investigation sought to evaluate the associations of (1) sport participation, and (2) single and multisport participation on physical fitness in American youth.

## 2. Materials and Methods

### 2.1. Participants

A secondary analysis was conducted using publicly available data from the National Youth Fitness Survey (NNYFS). The NNYFS was performed in conjunction with the National Health and Nutrition Examination Survey (NHANES) as a mode of addressing the need for physical activity and fitness data in children and adolescents. Although some measures were limited to select age demographics, the NNYFS overall included American youth aged 3–15 years. Interviews and fitness assessments were completed in various locations throughout the United States in the NHANES mobile examination centers. Similar to NHANES, the NNYFS utilized a multistage probability design for generating population-representative data for American youth.

The analytic sample included 1108 Americans aged 6–15 years with muscular fitness measures. This age range inclusion criteria were selected because muscular, cardiorespiratory endurance, and cardiovascular fitness measures were available for these ages. Trained interviewers collected self-report and physical measurements. Proxy respondents (e.g., parent) were used for questionnaires in those aged <12 years. Procedural reminders prior to any data entry were part of the quality control provisions for each assessment. Interview and examined response rates were >76% [17]. More details about the NNYFS are available elsewhere [18]. The NNYFS protocols were approved by the National Center for Health Statistics Research Ethics Review Board (Protocol 2011–20) and appropriate written informed consent from parents and guardians was provided.

### 2.2. Measures

The NNYFS includes three separate isometric muscular fitness assessments: handgrip strength, knee extension strength, and plank [19]. Briefly, participants removed any hand or wrist jewelry before completing standardized warm-up exercises to loosen up the hands and fingers. Interviewers fit the Takei T.K.K.5401 digital handgrip dynamometer (Takei Scientific Instruments; Niigata-City, Japan) to the hand size of each participant. Thereafter, participants completed a practice trial while standing with their feet hip with apart and arms fully extended. Participants squeezed the dynamometer with maximal force for three trials on each hand. The highest recorded handgrip strength was included in the analyses [20]. More details about the handgrip strength procedures in the NNYFS are available elsewhere [21].

Leg extension strength was measured with a Hoggan MicroFET2 (Hoggan Scientific; Salt Lake City, UT, USA) digital handheld dynamometer. Participants removed shoes, thick socks, and any ankle jewelry before completing standardized warm-up exercises for the lower extremities. Each person was seated in a chair specifically designed for NNYFS assessments which included straps to secure body position. A practice trial was allowed for familiarization. Interviewers positioned themselves and placed the dynamometer at ankle level. Participants pushed against the dynamometer with maximal effort for three trials on each leg. The highest recorded leg extension strength measurement was included in the analyses. More details about the leg extension strength protocols in the NNYFS are published elsewhere [22].

For the plank position assessment, participants began lying prone and then elevated themselves with their forearms and toes on the mat, and trunk balanced in a straight position. Participants were instructed to hold the plank position for as long as possible. We included the number of seconds the plank position was maintained in the analyses. More details about the plank hold assessment in the NNYFS are available elsewhere [23].

The NNYFS uses age-specific criteria for examining cardiorespiratory endurance. Trained interviewers explained and demonstrated the use of the Cardiac Science Quinton TM-55 treadmill (Bothell, WA, USA) before testing. Participants also walked on the treadmill for familiarization. For participants aged 6–11 years, an age-specific treadmill protocol that gradually increased in speed and grade was used. Each participant was asked to remain on the treadmill until voluntary exhaustion. The amount of time on the treadmill was included in the analyses.

For those aged 12–15 years, participants were assigned to one of five walking treadmill test protocols that varied in speed and grade based on their sex, age, body mass index, and self-report physical activity levels. Each protocol included a warm-up, two exercise periods, and cool down. Heart rate was monitored with four electrodes connected to the thorax and abdomen of participants, and the goal of each treadmill protocol was to elicit a heart rate that was approximately 75% of the age predicted maximum (220 minus age) by the end of testing. The estimated maximal oxygen uptake was included in the analyses. More details about the cardiorespiratory fitness procedures, including the equation used to estimate maximal oxygen uptake, used in the NNYFS are available elsewhere [24].

Interviewers asked respondents if they participate in school sports or physical activity clubs. Persons indicating that they engage in school sports or physical activity clubs told interviewers if they participate in the following activities: baseball, basketball, cheer, gymnastics, swimming, track-and-field, wrestling, dance, running, trampoline, martial arts, walking, hockey, frisbee, football, lacrosse, soccer, tennis, bocce ball, volleyball, or golf. Respondents suggesting participation in ≥2 activities as part of sports or clubs were considered as engaging in multiple sports.

Participants told interviewers about their age, sex, and race. Reported annual family income was used to determine family income to poverty ratio. A ratio of family income to poverty <1.0 was considered as living in poverty [25]. Respondents also told interviewers how many days they were physically active for a total of at least 60 min/day in the last week wherein physical activities increased heart and respiration rate (i.e., moderate-to-vigorous physical activity). Persons reporting engaging in such activities everyday were considered as meeting youth physical activity guidelines [26]. Standing height was measured with a SECA 217 portable stadiometer (SECA; Hamburg, Germany) and body mass was collected with a SECA 896 scale (SECA; Hamburg, Germany) using standard procedures [27]. Obesity was determined using the United States Centers for Disease Control and Prevention body mass index for age growth charts, wherein participants with a body mass index ≥95th percentile were obese [28].

### 2.3. Statistical Analysis

All analyses were performed with SAS 9.4 software (SAS Institute; Cary, NC, USA). The descriptive characteristics of the participants were presented as mean ± standard deviation for continuous variables and frequency (percentage) for categorical variables. The means and 95% confidence intervals (CI) for the descriptive characteristics were also shown as supplementary to make comparisons across any groups. Individual linear regression models analyzed the associations of any sports participation (reference: no sports participation) on (1) handgrip strength, (2) leg extension strength, (3) plank position held, (4) graded exercise test endurance, and (5) estimated maximal oxygen uptake. Separate linear regression models similarly evaluated the associations of (1) single sport participation, and (2) multisport participation (reference: no sports participation) on each marker of physical fitness. The linear regression models were adjusted for age, sex, race, meeting daily physical activity guidelines, obesity status, and poverty status. These covariates were pre-specified by the investigators and informed by previous work because they were thought to be influential for our associations [29].

Additionally, we provided the median (interquartile range) for the continuous descriptive characteristics of the participants as supplementary. Similarly, each of the response variables were log-transformed and included in the same linear regression modeling procedures from our primary analyses as another supplementary analysis. Further, each linear regression model from our primary analysis was stratified by gender. These additional analyses were presented as supplementary and thereby not discussed. An alpha level of 0.05 was used for all analyses.

## 3. Results

The descriptive characteristics of the participants are presented in Table 1. Overall, participants were aged 10.4 ± 2.8 years. Supplementary Table S1 shows the means and 95% CI for the descriptive characteristics by sport participation status, while Supplementary Table S2 presents the medians and interquartile ranges for the continuous characteristics by sport participation status. The descriptive characteristics by multisport participation status are in Table 2. Persons participating in no sports, a single sport, and multiple sports were aged 10.1 ± 2.9, 10.7 ± 2.8, and 10.6 ± 2.6 years, respectively. The means and 95% confidence intervals of the characteristics by multisport participation status are in Supplementary Table S3, whereas the medians and interquartile ranges for these continuous characteristics are shown in Supplementary Table S4.

Table 3 presents the results for the associations between any sports participation status and markers of physical fitness. Youth participating in any sports had increased performance for each physical fitness marker compared to persons not engaging in sports. Specifically, handgrip strength increased 1.63 kg (CI: 0.91, 2.35; *p* < 0.001), leg extension strength increased 6.23 kg (CI: 3.15, 9.32; *p* < 0.001), plank position held increased 9.41 s (CI: 4.51, 14.31; *p* < 0.001), and estimated maximal oxygen uptake increased 3.02 mL/kg/min (CI: 1.23, 4.80; *p* < 0.001). The associations between multisport participation status and markers of physical fitness are shown in Table 4. Relative to no sport participation, youth engaging in a single sport had increased performance for most physical fitness markers: 1.52 kg (CI: 0.67, 2.38; *p* < 0.001) for handgrip strength, 5.16 kg (CI: 1.50, 8.82; *p* = 0.005) for leg extension strength, 5.98 s (CI: 0.17, 11.78; *p* = 0.04) for plank position held, 2.67 mL/kg/min (CI: 0.58, 4.77; *p* = 0.01) for estimated maximal oxygen uptake. Likewise, compared to no sport participation, a similar trend was observed in youth engaging in multiple sports, but the magnitude of performance on the physical fitness tests was steeper: 1.77 kg (CI: 0.83, 2.70; *p* < 0.001) for handgrip strength, 7.63 kg (CI: 3.62, 11.65; *p* < 0.001) for leg extension strength, 13.89 s (CI: 7.52, 20.27; *p* < 0.001) for plank position held, and 3.50 mL/kg/min (CI: 1.15, 5.85; *p* = 0.003) for estimated maximal oxygen uptake. No significant associations were observed for single or multisport participation on graded exercise test endurance.

The associations between any sports participation status and log transformed markers of physical fitness are in Supplementary Table S5, while the log transformed markers of physical fitness for multisport participation status are presented in Supplementary Table S6. The log transformed findings were compatible with our principal findings. Moreover, the gender stratified results for the associations between any sports and multisport participation for physical fitness are shown in Supplementary Tables S7 and S8, respectively.

## 4. Discussion

The principal results of this investigation revealed an association between any sports participation and increased physical fitness in younger Americans. Further, both single and multisport engagement was associated with increased physical fitness, even after controlling for relevant covariates. Our findings indicate that youth sports participation is linked to increased musculoskeletal and cardiorespiratory physical fitness. We recommend participation in multiple sports for physical fitness when possible. Physical educators, parents/guardians, coaches, and younger populations should have awareness of the fitness-related benefits associated with any sports involvement.

Findings from previous work support youth sport participation for elevating physical activity participation [5], and our findings are in alignment with such work wherein sport-related physical activity may help to increase physical fitness. Although we revealed any sports participation is associated with muscular and cardiorespiratory fitness, the majority of our included participants did not engage in sports. Several recommendations have been provided to encourage youth sport participation for increasing physical activity such as providing access to sport-related before and after school programs, and allow sharing of sports facilities in schools for community-based organizations [30].

Physical educators may also include sport activities as part of the regular physical education program to address various learning standards. However, age, sport classifications (e.g., fielding/striking games, invasion games, individual sport), modifications for individual developmental levels, and student choice should be carefully considered while using best practices to ensure youth have enjoyable sporting experiences [31,32]. Such experiences should provide opportunities for youth to develop the knowledge, skills, and confidence to participate in sports outside of the regular physical education programming [33]. Physical education may similarly consider instructional models such as Sport Education and Tactical Games for Understanding given their positive impact on enjoyment and motivation to engage in sport experience [34,35,36]. Likewise, youth coaches should follow similar recommendations to ensure positive sporting experiences in order to foster continued participation in sports [37]. Nonetheless, sport participation should be supported as a strategy for increasing physical fitness and health in youth.

While participation in any sport may increase physical fitness, youth participating in multiple sports may experience elevated fitness levels. Popović et al. [38]. showed that youth who participated in a 9-month structured multisport program had increased their standing long jump and core strength compared to an exercise program control. Engagement with a more diverse set of motor skills inherent with participating in multiple sports could lead to advancements in motor competence [39,40], which is a predictor of physical activity engagement [41], and thereby fitness. While the results of this study indicate that both single and multisport participation were associated with increased handgrip strength, leg extension strength, plank position held, and estimated maximal oxygen uptake, the magnitude of these associations was steeper in multisport participants. Although there could be many factors that contribute to why multisport participation is particularly beneficial for physical fitness, our findings nevertheless support multisport participation for youth when possible.

Youth sport participation remains common in the United States and has maintained a higher achieving indicator for the United States Youth Physical Activity Report Card [5]. Continuing to help youth circumvent barriers to sport participation should be encouraged. School physical education classes could be an excellent resource for helping less skilled youth develop their competency with a focus on individualized improvement [42]. Providing activity choice and relatedness alongside such competency may improve self-determination to engage in potentially several sports [43,44]. Helping youth achieve comfort with sport participation may also help with lifespan activity adherence, thereby combating health conditions related to sedentary behavior. Future research may examine how different didactic methods in physical education classes stimulate sport interest and participation in non-active youth.

Some limitations should be noted. The NNYFS utilizes a cross-sectional design and inherent limitations may exist for direction of the associations in our study. Robust trials with repeated measures will help to support or dispute our findings. Data availability limited which covariates could be included in our models. Although self-report data are common in epidemiological studies such as the NNYFS, biases related to self-report may have influenced our findings. While our findings showed that any sports participation may increase physical fitness, examining the role of sports types may reveal additional insights into how sports impact fitness. The NNYFS includes data for a national sample of young Americans, but these data are not nationally-representative. Likewise, NNYFS data are not generalizable to other countries. Despite these limitations, our investigation included NNYFS data, which included larger sample of younger Americans. The protocols for the measures in the NNYFS were also reliable, valid, and standardized.

## 5. Conclusions

Our investigation revealed that participation in any sport, when compared to no sports participation, was associated with increased muscular and cardiorespiratory fitness in younger Americans. Similarly, single and multisport participation was associated with increased markers of physical fitness. Our findings should be used to inform parents/guardians, physical educators, coaches, and youth about the health benefits related to sports participation, and how these benefits could be potentiated when multisport participation is present. We recommend that resources be provided for helping youth overcome barriers related to sport participation. Overcoming such barriers may help American youth improve their fitness, maintain activity participation, and reduce the risk for health conditions related to physical inactivity.

## Figures and Tables

**Table 1:** Descriptive Characteristics of the Participants.

Variable	Overall (*n* = 1108)	Participates in Sports (*n* = 508)	No Sports Participation (*n* = 600)
Age (years)	10.4 ± 2.8	10.7 ± 2.7	10.1 ± 2.9
Aged 12–15 Years	445 (40.1)	210 (41.3)	235 (39.1)
Female (n (%))	556 (50.1)	239 (47.0)	317 (52.8)
Obese (n (%))	223 (20.1)	98 (19.2)	125 (20.8)
White Race (n (%))	448 (40.4)	216 (42.5)	232 (38.6)
Income-to-Poverty Ratio	2.3 ± 1.6	2.4 ± 1.6	2.2 ± 1.6
Below Poverty Threshold (n (%))	324 (29.2)	125 (24.6)	199 (33.1)
Meeting Youth Physical Activity Guidelines (n (%))	509 (45.9)	237 (46.6)	272 (45.3)
Handgrip Strength (kilograms)	22.3 ± 9.6	23.4 ± 9.7	21.4 ± 9.5
Leg Extension Strength (kilograms)	62.8 ± 33.2	66.7 ± 33.8	59.6 ± 32.3
Plank Position Held (seconds)	68.9 ± 45.3	75.1 ± 46.7	63.8 ± 43.5
Graded Exercise Test Endurance Time (seconds) †	650.5 ± 135.3	655.4 ± 136.5	646.6 ± 134.4
Estimated Maximal Oxygen Uptake (mL/kg/min) ‡	41.3 ± 9.8	43.0 ± 10.1	39.8 ± 9.3

† *n* = 631 overall aged 6–11 years (*n* = 352 no sports participation; *n* = 279 sports participation). ‡ *n* = 417 overall aged 12–15 years (*n* = 219 no sports participation; *n* = 198 sports participation). Note: mL/kg/min = milliliters per kilograms per minute.

**Table 2:** Descriptive Characteristics of the participants by Multisport Participation Status.

Variable	No Sports Participation (*n* = 600)	Single Sport Participation (*n* = 287)	Multisport Participation (*n* = 221)
Age (years)	10.1 ± 2.9	10.7 ± 2.8	10.6 ± 2.6
Aged 12–15 Years	235 (39.1)	122 (42.5)	88 (39.8)
Female (n (%))	317 (52.8)	141 (49.1)	98 (44.3)
Obese (n (%))	125 (20.8)	59 (20.5)	39 (17.6)
White Race (n (%))	232 (38.6)	124 (43.2)	92 (41.6)
Income-to-Poverty Ratio	2.2 ± 1.6	2.4 ± 1.6	2.4 ± 1.6
Below Poverty Threshold (n (%))	199 (33.1)	71 (24.7)	54 (24.4)
Meeting Youth Physical Activity Guidelines (n (%))	272 (45.3)	130 (45.3)	107 (48.2)
Handgrip Strength (kilograms)	21.4 ± 9.5	23.4 ± 10.0	23.3 ± 9.3
Leg Extension Strength (kilograms)	59.6 ± 32.3	66.2 ± 35.5	67.2 ± 31.5
Plank Position Held (seconds)	63.8 ± 43.5	71.3 ± 43.7	79.9 ± 50.0
Graded Exercise Test Endurance Time (seconds) †	646.6 ± 134.4	651.2 ± 127.3	660 ± 147.1
Estimated Maximal Oxygen Uptake (mL/kg/min) ‡	39.8 ± 9.3	42.1 ± 9.9	44.2 ± 10.3

† *n* = 352 no sport participation, *n* = 151 single sport participation, *n* = 128 multisport participation. ‡ *n* = 219 no sports participation, *n* = 115 single sport participation, *n* = 83 multisport participation. Note: mL/kg/min = milliliters per kilograms per minute.

**Table 3:** Associations Between Any Sports Participation Status and Markers of Physical Fitness.

Variable	Participates in Sport		
	β	95% Confidence Interval	*p*-Value
Handgrip Strength	1.63	0.91, 2.35	<0.001
Leg Extension Strength	6.23	3.15, 9.32	<0.001
Plank Position Held	9.41	4.51, 14.31	<0.001
Graded Exercise Test Endurance	4.22	−15.25, 23.71	0.67
Estimated Maximal Oxygen Uptake	3.02	1.23, 4.80	0.001

Reference: no sports participation. Note: each model was adjusted for daily physical activity participation, sex, age, obesity status, race, and poverty status.

**Table 4:** Associations Between Multisport Participation Status and Markers of Physical Fitness.

Variable	Single Sport Participation			Multisport Participation		
	β	95% CI	*p*-Value	β	95% CI	*p*-Value
Handgrip Strength	1.52	0.67, 2.38	<0.001	1.77	0.83, 2.70	<0.001
Leg Extension Strength	5.16	1.50, 8.82	0.005	7.63	3.62, 11.65	<0.001
Plank Position Held	5.98	0.17, 11.78	0.04	13.89	7.52, 20.27	<0.001
Graded Exercise Test Endurance	1.75	−21.89, 25.39	0.88	7.14	−17.92, 32.21	0.57
Estimated Maximal Oxygen Uptake	2.67	0.58, 4.77	0.01	3.50	1.15, 5.85	0.003

Reference: no sports participation. Note: each model was adjusted for daily physical activity participation, sex, age, obesity status, race, and poverty status.

## Data Availability

National Health and Nutrition Examination Survey National Youth Fitness Survey data are publicly available online: https://www.cdc.gov/nchs/nnyfs/index.htm.

## Supplementary Materials

The following supporting information can be downloaded at: https://www.mdpi.com/article/10.3390/sports10120202/s1, Table S1: Means and 95% Confidence Intervals for the Descriptive Characteristics by Sport Participation Status; Table S2: Medians and Interquartile Ranges for the Continuous Characteristics by Sport Participation Status; Table S3: Means and 95% Confidence Intervals for the Characteristics by Multisport Participation Status; Table S4: Medians and Interquartile Ranges for the Continuous Characteristics by Sport Participation Status; Table S5: Associations Between Any Sports Participation Status and Log Transformed Markers of Physical Fitness; Table S6: Associations Between Multisport Participation Status and Log Transformed Markers of Physical Fitness; Table S7: Associations Between Any Sports Participation Status and Markers of Physical Fitness by Gender; Table S8: Associations Between Multisport Participation Status and Markers of Physical Fitness by Gender.
