# Supplementary material for: Single and Especially Multisport Participation May Increase Physical Fitness in Younger Americans
Source: Sports (Basel). 2022 Dec 9;10(12):202. doi: 10.3390/sports10120202 (PMC9786319; doi:10.3390/sports10120202)
Supplement: Supplementary file 1 [file sports-10-00202-s001.zip › sports-2003881-supplementary.pdf]

Supplementary Materials

Table S1. Means and 95% Confidence Intervals for the Descriptive Characteristics by Sport Participation Status.

Variable	Participates in Sports	No Sports Participation
Age (years)	10.7 (10.4, 10.9)	10.1 (9.9, 10.4)
Aged 12-15 Years	41.3 (37.0, 45.6)	39.1 (35.2, 43.0)
Female (n (%))	47.0 (42.7, 51.3)	52.8 (48.8, 56.8)
Obese (n (%))	19.2 (15.8, 22.7)	20.8 (17.5, 24.0)
White Race (n (%))	42.5 (38.2, 46.8)	38.6 (34.7, 42.5)
Income-to-Poverty Ratio	2.4 (2.2, 2.5)	2.2 (2.0, 2.3)
Below Poverty Threshold (n (%))	24.6 (20.8, 28.3)	33.1 (29.4, 36.9)
Meeting Youth Physical Activity Guidelines (n (%))	46.6 (42.3, 50.9)	45.3 (41.3, 49.3)
Handgrip Strength (kilograms)	23.4 (22.5, 24.6)	21.4 (20.6, 22.2)
Leg Extension Strength (kilograms)	66.7 (63.7, 69.6)	59.6 (57.0, 62.2)
Plank Position Held (seconds)	75.1 (71.0, 79.1)	63.8 (60.3, 67.2)
Graded Exercise Test Endurance Time (seconds)	655.4 (639.3, 671.5)	646.6 (632.5, 660.7)
Estimated Maximal Oxygen Uptake (ml/kg/min)	43.0 (41.6, 44.4)	39.8 (38.5, 41.0)

Table S2. Medians and Interquartile Ranges for the Continuous Characteristics by Sport Participation Status.

Variable	Participates in Sports	No Sports Participation
Age (years)	11.0 (8.0, 13.0)	10.0 (7.0, 13.0)
Income-to-Poverty Ratio	2.0 (1.0, 3.8)	1.7 (0.7, 3.5)
Handgrip Strength (kilograms)	22.1 (15.7, 29.6)	19.6 (13.8, 26.9)
Leg Extension Strength (kilograms)	61.3 (40.8, 87.4)	51.8 (34.9, 79.3)
Plank Position Held (seconds)	67.0 (42.0, 97.5)	60.0 (33.0, 84.0)
Graded Exercise Test Endurance Time (seconds)	657.0 (570.0, 737.0)	662.0 (556.0, 741.0)
Estimated Maximal Oxygen Uptake (ml/kg/min)	40.9 (35.9, 48.6)	37.8 (33.4, 44.3)

Table S3. Means and 95% Confidence Intervals for the Characteristics by Multisport Participation Status.

Variable	No Sports Participation	Single Sport Participation	Multisport Participation
Age (years)	10.1 (9.9, 10.4)	10.7 (10.4, 11.0)	10.6 (10.3, 10.9)
Aged 12-15 Years	39.1 (35.2, 43.0)	42.5 (36.7, 48.2)	39.8 (33.3, 46.2)
Female (n (%))	52.8 (48.8, 56.8)	49.1 (43.3, 54.9)	44.3 (37.7, 50.8)
Obese (n (%))	20.8 (17.5, 24.0)	20.5 (15.8, 25.2)	17.6 (12.6, 22.6)
White Race (n (%))	38.6 (34.7, 42.5)	43.2 (37.4, 48.9)	41.6 (35.1, 48.1)
Income-to-Poverty Ratio	2.2 (2.0, 2.3)	2.4 (2.2, 2.5)	2.4 (2.2, 2.6)
Below Poverty Threshold (n (%))	33.1 (29.4, 36.9)	24.7 (19.7, 29.7)	24.4 (18.7, 30.1)
Meeting Youth Physical Activity Guidelines (n (%))	45.3 (41.3, 49.3)	45.3 (39.5, 51.0)	48.2 (41.8, 55.0)
Handgrip Strength (kilograms)	21.4 (20.6, 22.2)	23.4 (22.3, 24.6)	23.3 (22.1, 24.5)
Leg Extension Strength (kilograms)	59.6 (57.0, 62.2)	66.2 (62.1, 70.3)	67.2 (63.1, 71.4)
Plank Position Held (seconds)	63.8 (60.3, 67.2)	71.3 (66.3, 76.4)	79.9 (73.3, 86.6)
Graded Exercise Test Endurance Time (seconds)	646.6 (632.5, 660.7)	651.2 (630.8, 671.7)	660.4 (634.6, 686.1)
Estimated Maximal Oxygen Uptake (ml/kg/min)	39.8 (38.5, 41.0)	42.1 (40.3, 44.0)	44.2 (41.9, 46.4)

Table S4. Medians and Interquartile Ranges for the Continuous Characteristics by Sport Participation Status.

Variable	No Sports Participation	Single Sport Participation	Multisport Participation
Age (years)	10.0 (7.0, 13.0)	11.0 (8.0, 13.0)	11.0 (8.0, 13.0)
Income-to-Poverty Ratio	1.7 (0.7, 3.5)	1.9 (1.0, 3.7)	2.1 (1.0, 3.9)
Handgrip Strength (kilograms)	19.6 (13.8, 26.9)	22.3 (15.4, 30.3)	21.8 (16.4, 28.9)
Leg Extension Strength (kilograms)	51.8 (34.9, 79.5)	60.3 (39.1, 87.6)	61.8 (44.5, 87.3)
Plank Position Held (seconds)	60.0 (33.0, 84.0)	65.0 (41.0, 95.0)	69.0 (45.0, 101.0)
Graded Exercise Test Endurance Time (seconds)	662.0 (556.0, 741.0)	656.0 (559.0, 733.0)	659.0 (577.5, 756.0)
Estimated Maximal Oxygen Uptake (ml/kg/min)	37.8 (33.4, 44.3)	39.2 (35.6, 46.9)	42.9 (37.1, 50.0)

Table S5. Associations Between Any Sports Participation Status and Log Transformed Markers of Physical Fitness.

Variable	Participates in Sport		
	β	95% Confidence Interval	p-value
Handgrip Strength	0.08	0.05, 0.11	<0.001
Leg Extension Strength	0.11	0.06, 0.16	<0.001
Plank Position Held	0.16	0.07, 0.25	<0.001
Graded Exercise Test Endurance	0.01	-0.03, 0.04	0.78
Estimated Maximal Oxygen Uptake	0.07	0.03, 0.11	<0.001

Table S6. Associations Between Multisport Participation Status and Log Transformed Markers of Physical Fitness.

Variable	Single Sport Participation			Multisport Participation		
	β	95% CI	p-value	β	95% CI	p-value
Handgrip Strength	0.07	0.03, 0.10	<0.001	0.09	0.05, 0.14	<0.001
Leg Extension Strength	0.08	0.01, 0.14	0.01	0.15	0.08, 0.22	<0.001
Plank Position Held	0.11	0.01, 0.21	0.02	0.23	0.11, 0.34	<0.001
Graded Exercise Test Endurance	0.01	-0.03, 0.06	0.60	-0.01	-0.05, 0.05	0.90
Estimated Maximal Oxygen Uptake	0.06	0.01, 0.11	0.005	0.08	0.03, 0.13	0.001

Table S7. Associations Between Any Sports Participation Status and Markers of Physical Fitness by Gender.

Variable	Participates in Sport		
	β	95% Confidence Interval	p-value
Males			
Handgrip Strength	1.90	0.77, 3.04	0.001
Leg Extension Strength	7.31	2.54, 12.08	0.002
Plank Position Held	11.58	4.17, 18.98	0.002
Graded Exercise Test Endurance	19.04	-11.39, 49.48	0.21
Estimated Maximal Oxygen Uptake	4.44	1.93, 6.96	<0.001
Females			
Handgrip Strength	1.42	0.61, 2.23	<0.001
Leg Extension Strength	5.36	1.49, 9.23	0.006
Plank Position Held	7.50	1.12, 13.88	0.02
Graded Exercise Test Endurance	-7.26	-31.73, 17.20	0.55
Estimated Maximal Oxygen Uptake	1.49	-1.06, 4.05	0.25

Table S8. Associations Between Multisport Participation Status and Markers of Physical Fitness by Gender.

Variable	Single Sport Participation			Multisport Participation		
	β	95% CI	p-value	β	95% CI	p-value
Males						
Handgrip Strength	1.84	0.48, 3.20	0.007	1.98	0.53, 3.42	0.007
Leg Extension Strength	6.69	0.99, 12.40	0.02	8.05	1.99, 14.12	0.009
Plank Position Held	6.78	-2.03, 15.61	0.13	17.33	7.94, 26.71	<0.001
Graded Exercise Test Endurance	15.50	-20.99, 52.01	0.40	23.25	-15.47, 61.98	0.23
Estimated Maximal Oxygen Uptake	3.59	0.52, 6.65	0.02	5.32	2.24, 8.40	<0.001
Females						
Handgrip Strength	1.50	0.54, 2.47	0.002	1.30	0.21, 2.39	0.01
Leg Extension Strength	4.35	-0.22, 8.94	0.06	6.78	1.58, 11.97	0.01
Plank Position Held	6.12	-1.44, 13.69	0.11	9.43	0.86, 18.01	0.03
Graded Exercise Test Endurance	-5.85	-36.07, 24.37	0.70	-8.89	-40.81, 23.01	0.58
Estimated Maximal Oxygen Uptake	1.75	-1.14, 4.64	0.23	0.98	-2.72, 4.70	0.60
